# Upstream therapeutic strategies of Valsartan and Fluvastatin on Hypertensive patients with non-permanent Atrial Fibrillation (VF-HT-AF): study protocol for a randomized controlled trial

**DOI:** 10.1186/s13063-015-0836-5

**Published:** 2015-08-07

**Authors:** Wen-Wei Qi, Tong Liu, Gang Xu, Li-Feng Li, Ying-Zi Liang, Lan Ye, Guang-Ping Li

**Affiliations:** Tianjin Key Laboratory of Ionic-Molecular Function of Cardiovascular disease, Department of Cardiology, Tianjin Institute of Cardiology, Second Hospital of Tianjin Medical University, Tianjin, 300211 PR China

**Keywords:** 7-day Holter, Atrial fibrillation, Fluvastatin, Randomized controlled clinical trial, Valsartan

## Abstract

**Background:**

Previous studies regarding rhythm control in patients with atrial fibrillation (AF) could not sufficiently demonstrate the efficacy of available anti-arrhythmic drugs. ‘Upstream therapy’ has emerged as a potential strategy for the prevention and treatment of AF. The use of angiotensin II receptor blockers and statins has been suggested to decrease new-onset AF, but which remains inadequately explored. This study was designed to examine whether valsartan or fluvastatin can reduce the risk of non-permanent AF in patients with hypertension.

**Methods/design:**

The VF-HT-AF study is a multicenter, randomized, open-label, four-arm parallel group study with comparative evaluation of valsartan and fluvastatin as upstream therapies for the treatment of non-permanent AF complicated by hypertension. The primary outcome measure is change in the development of paroxysmal AF into persistent or permanent AF, the development of persistent AF to permanent AF, and change in incidence of overall and persistent AF recurrence, as evaluated by 7-days ambulatory electrocardiograph monitoring (Holter) and patients’ diaries during 2 years’ follow-up. Secondary outcome measures of this study include the occurrence of: (1) fatal and nonfatal myocardial infarction; (2) heart failure (New York Heart Association stage III or IV); (3) cardiogenic shock; (4) serious bleeding necessitating hospitalization; (5) malignant ventricular arrhythmia; (6) revascularization therapy; (7) radiofrequency catheter ablation of AF; (8) changes of left atrial dimension, as measured by ultrasound echocardiography; (9) stroke; (10) cardiovascular mortality; and (11) all-cause mortality. A total of 1879 patients will be investigated from 15 medical centers throughout China to obtain the relevant information.

**Discussion:**

This is the first study in hypertensive patients complicated non-permanent AF in the Chinese population. Results of this study will inform the use of upstream therapies of AF.

**Trial registration:**

chictr.org, ChiCTR-TRC-12002642

**Electronic supplementary material:**

The online version of this article (doi:10.1186/s13063-015-0836-5) contains supplementary material, which is available to authorized users.

## Background

Atrial fibrillation (AF), the most common clinically significant cardiac arrhythmia, is associated with increased mortality and morbidity [1, 2], especially in hypertensive patients [3]. In general, hypertension is the most important risk factor for AF. Although much effort has been put into the development of an effective pharmacological treatment, several trials conducted in the USA and Europe proved that existing traditional anti-arrhythmic drugs failed to improve the prognosis of patients with AF [4–7].

Several systematic reviews and meta-analyses have confirmed anti-arrhythmic efficacy while raising concerns about adverse events and mortality [8]. In the past few years, a number of trials investigating upstream therapy for the prevention of AF have been reported [9]. Upstream therapies are long-term modulators of atrial remodeling regarding structure or function, which may change molecular expression contributing to the arrhythmia. The renin-angiotensin system (RAS) is an important therapeutic target for atrial remodeling. Angiotensin II receptor blockers (ARBs) have recently been reported to suppress AF recurrence in both paroxysmal and persistent AF in selected patients. However, recent placebo-controlled, double-blind trials with ARBs failed to show convincing results [10, 11]. Statins, as a well-established secondary prevention benefit for atherosclerotic coronary artery disease, are hypothesized to be beneficial against atrial arrhythmia, yet the data are inconsistent [12–15]. In addition, there have been little prospective and randomized studies to evaluate the effectiveness of upstream therapeutic strategies in hypertensive patients with non-permanent AF.

To confirm the effectiveness of ARBs or statins on AF in hypertensive patients, the Upstream Therapeutic Strategies of Valsartan and Fluvastatin on Hypertensive Patients with Non-permanent AF (VF-HT-AF study) will be conducted, with 1879 participants from 15 centers.

## Methods/design

The VF-HT-AF study is designed as a prospective, randomized, open-label, four-arm parallel, and multicenter study. The objective of the study is to test the hypothesis that upstream therapy using valsartan or fluvastatin is more effective in reducing the recurrence of AF and the progress from non-permanent AF to permanent AF in hypertensive patients with non-permanent AF, compared with conventional antihypertensive therapy using dihydropyridine calcium channel blockers (CCBs). This study will be conducted according to the principles outlined in the Declaration of Helsinki. Written informed consent will be obtained from all patients prior to the study. The study protocol has been approved by the ethics committee of the Second Hospital of Tianjin Medical University (Clinical ethical review, 2012, No. 27) as well as other participating medical centers, which are secondary or tertiary healthcare providers in China, and should provide proof of laboratory quality control. The ethical bodies that approved our study in the various centers are listed in Additional file 1. If there is any amendment to the protocol, approval must again be sought from the ethics committee. The study is registered with the Chinese Clinical Trial Registry (ChiCTR-TRC-12002642). The protocol design is based on the Consolidated Standards of Reporting Trials (CONSORT) and Standard Protocol Items: Recommendations for Interventional Trials (SPIRIT, see Additional file 2), and study results will be reported according to these guidelines.

### Patient population and entry criteria

Patients enrolled in the study need to meet the inclusive recruited criteria: (1) have hypertension, defined as an average systolic blood pressure ≥140 mmHg (1 mm Hg = 0.133 kPa) or a diastolic blood pressure ≥90mmHg (but a systolic blood pressure <180 mmHg and a diastolic blood pressure <110 mmHg) at the first visit, or the requirement of any anti-hypertension treatment at enrollment; (2) a history of non-permanent AF within 1 year prior to the enrollment, as confirmed by electrocardiography (ECG), and converted to sinus rhythm; (3) have not taken ARBs or angiotensin-converting enzyme inhibitors (ACEIs) as well as statins in the previous 2 weeks, or are taking ARBs, ACEIs and statins, but can accept a 2-week washout period; (4) aged 25–79 years; and (5) willing to sign the informed consent form (Additional file 3).

The exclusion criteria are: (1) persistent AF with a duration ≥1 year or permanent AF; (2) serious left main coronary artery disease identified by coronary angiography; (3) heart failure (New York Heart Association [NYHA] stage III or IV); (4) acute myocardial infarction in the previous 3 months; (5) surgical or interventional indications of valvular heart disease; (6) uncontrolled thyroid disease (abnormal free T3, free T4, or thyroid stimulating hormone, or requiring any anti-thyroid treatment at enrollment); (7) serious liver or renal dysfunction (ALT > 80 U/l, AST > 80 U/l, or creatinine > 132 μmol/l); (8) history of unstable angina pectoris; (9) stroke or transient ischemic attack within the previous 3 months; (10) poor treatment compliance, such as central nervous system or mental illness, or a possibility of being uncooperative in the follow-up period; (11) have taken ARBs, ACEIs or statins but cannot accept a 2-week washout period; (12) obvious hyperlipidemia that must be treated by statins or fibrates; (13) contraindication of statins or ARBs; (14) pregnancy or the possibility of pregnancy, or breast feeding; and (15) aged younger than 25 or older than 79 years.

### Study design

The VF-HT-AF study is a randomized, multicenter, open-label, and four-arm parallel study to evaluate the effectiveness of valsartan, fluvastatin or a combination of both on recurrent AF in hypertensive patients with non-permanent AF in China. After providing informed consent, patients will be randomly assigned using a computer system to one of four groups (the valsartan group, the fluvastatin plus dihydropyridine CCBs group, the valsartan plus fluvastatin group, and the dihydropyridine CCBs group) by the School of Public Health of Tianjin Medical University, initially in a 1:1:1:1 ratio. Physicians, who should be qualified medical practitioners, screen and follow up the patients in hospital outpatient or inpatient clinics. All patients’ data will be collected using a standard case report form and transmitted to the central database at the data center. All centers will be regularly monitored for source data documentation. Missing or questionable data will be completed and corrected by queries.

If the candidate patients have not taken ARBs, ACEIs or statins in the past at least 14 days, they will be directly enrolled and randomized. If the candidate patients have taken ARBs, ACEIs or statins, they will be randomized after a 14-day washout period. Baseline data will be collected after the washout period.

The initial dose of fluvastatin will be 40 mg/day at night, while valsartan will be prescribed initially at 80 mg/day. The fluvastatin dose will be no less than 40 mg/day and that of valsartan will be no less than 80 mg/day during the follow-up period. Blood pressures in each group must reach the target blood pressure, which is set at <140/90 mm Hg.

### Follow-up

Clinical follow-up using standardized questionnaires will be performed every 3 months during the 2 years follow-up period from the assignment of each patient.

The dosages and types of all other anti-hypertensive, anti-hyperlipidemia, and anti-arrhythmic drugs used during the follow-up will be recorded for each patient. Ultrasound echocardiography will be performed before the patients enter the study and at the end of the follow-up period. 7-day Holter monitoring will be performed at the baseline, 6 month, 12 month and at the end of the follow-up period. Patients’ diaries, cardiac function (NYHA classification) and adverse events during the follow-up period will be collected every 3 months.

**Fig. 1:**
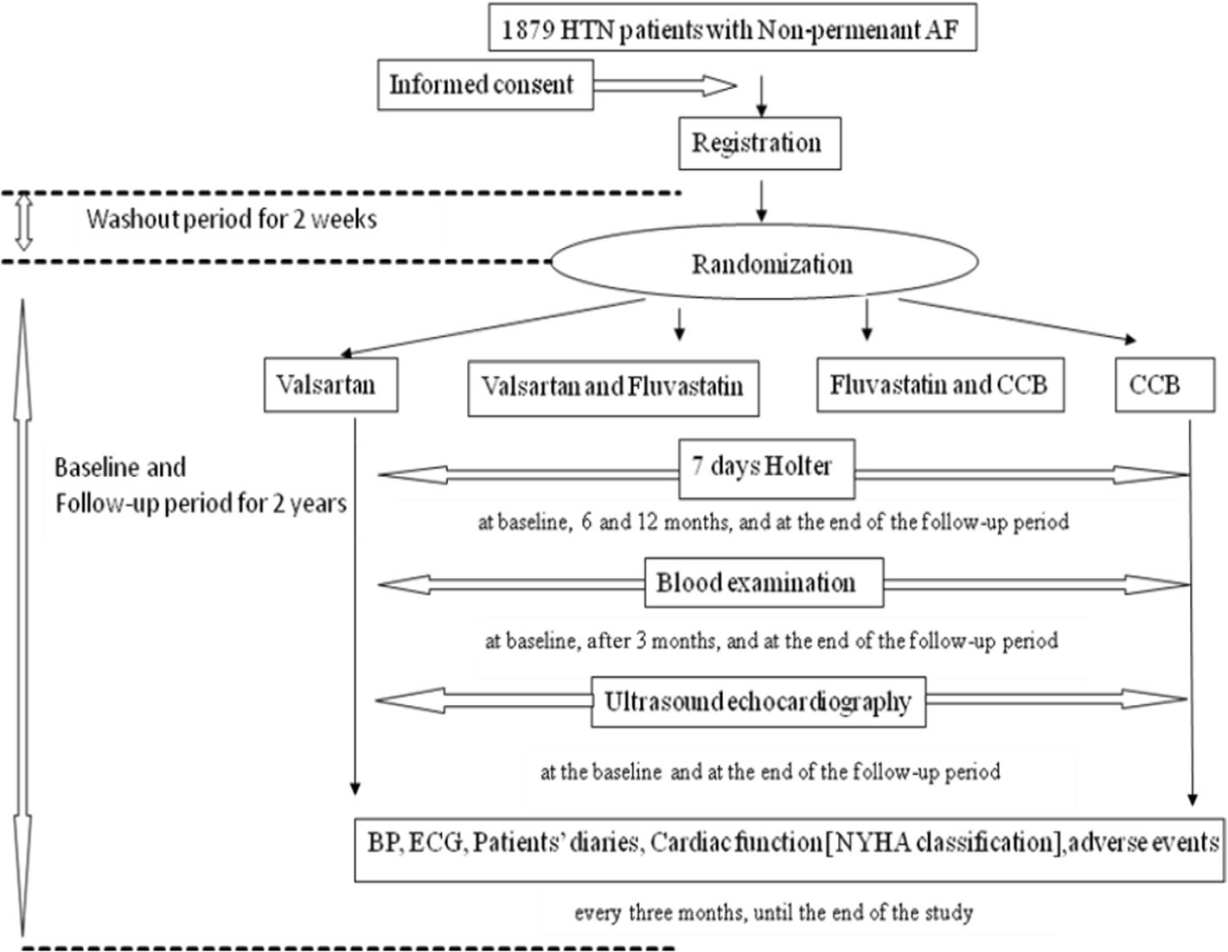
Flow-process diagram. BP, blood pressure; ECG, electrocardiography

Throughout the study, patients should record diaries when they feel discomfort. Arrhythmia-related symptoms are self-evaluated or evaluated by 7-day Holter monitoring, which has five poles, is weighted 50 g, 2.0 × 2.7 cm, and can record ECG for 7 days. The 7-day Holter instruments were manufactured by BORSAM Co. in Shenzhen, Guangdong Province, China, and provided by the study’s sponsor, Tianjin Institute of Cardiology, Tianjin, China. The attending physicians will also record blood pressure and ECG during each follow-up. Unless necessary, anti-arrhythmic drug therapy will be discontinued during the study according to the attending physicians’ advice for each patient. The design of the study is shown in Fig. 1.

### Compliance strategy

To maximize subjects’ compliance, first, we will have a thorough consent process for all participants. We will explain the schedule, potential side effects of treatment, and the responsibilities of the subjects in detail. Second, we will try to prevent dropouts by providing ongoing support to patients. A direct telephone line set up for this trial will enable the study team to communicate with the patients personally. An information sheet will be given to each participant providing them or their caregivers with means of urgent contact. Extra visits and free medical care will be arranged for any participant who feels harmed by the trial protocol. Certainly those participants in the control group should not experience any difference in their condition.

### Primary outcome measures

The VF-HT-AF study will examine whether fluvastatin or valsartan has anti-arrhythmic effects on non-permanent AF, apart from their lipid-lowering and antihypertensive effects, in comparison with dihydropyridine CCBs. Also, it will confirm whether a combination of valsartan and fluvastatin has better effects on non-permanent AF than either drug alone.

Therefore, the primary outcome measure is the change in the development of paroxysmal AF into persistent or permanent AF, the development of persistent AF to permanent AF, as well as the change in incidence of overall and persistent AF recurrence, which will be evaluated by 7-day Holter monitoring and patients’ diaries during the 2-year’s follow-up of the four treatment groups.

### Secondary outcome measures

The secondary outcome measures of this study include the occurrence of: (1) myocardial infarction; (2) heart failure (NYHA III or IV); (3) cardiogenic shock; (4) serious bleeding necessitating hospitalization; (5) malignant ventricular arrhythmia (including ventricular tachycardia or fibrillation); (6) revascularization therapy (coronary artery bypass graft or percutaneous coronary interventions); (7) radiofrequency catheter ablation of AF; (8) changes of left atrial dimension, as measured by ultrasound echocardiography; (9) stroke; (10) cardiovascular mortality; and (11) all-cause mortality.

### Definitions

The definitions of AF are made according to 2012 HRS/EHRA/ECAS Expert Consensus [16]. Paroxysmal AF is defined as recurrent AF (at least two episodes) that terminates spontaneously within 7 days. Episodes of AF no more than 48 hours’ duration that are terminated with electrical or pharmacologic cardioversion should also be classified as paroxysmal AF episodes. Persistent AF is defined as continuous AF that is sustained beyond 7 days. Episodes of AF lasting more than 48 hours, but less than 7 days, in which a decision is made to cardiovert AF electrically or pharmacologically should also be classified as persistent AF episodes. Permanent AF refers to long-lasting episodes of AF that could be considered by any evidence as lasting longer than 1 year, or to a case of AF for which a decision has been made not to restore or maintain sinus rhythm by any means, including catheter ablation or surgery. Myocardial infarction is defined as a cardiac troponin rise accompanied by typical symptoms, pathological Q waves, ST elevation or depression, or coronary intervention. Heart failure (NYHA III or IV) requires echocardiographic evidence of reduced left ventricular ejection fraction or a diagnosis of heart failure by a cardiologist. Cardiogenic shock is defined by sustained hypotension with tissue hypoperfusion despite adequate left ventricular filling pressure. Signs of tissue hypoperfusion include oliguria (<30 ml/hour), cool extremities, and altered level of consciousness.

### Sample size calculation

The primary outcome measure is the change in the development of paroxysmal AF into persistent or permanent AF, the development of persistent AF to permanent AF, as well as the change in incidence of overall and persistent AF recurrence. Power calculations to provide estimates for the necessary sample size were conducted concerning the primary outcome measure. The sample size was estimated based on findings from the 12-month results of the J-RHYTHM II study [11], in which the incidence of persistent AF is estimated to be 6.8 % lower in the valsartan group than in the CCBs group. To ensure 80 % power at the 5 % significance level, a sample size of at least 1404 needs to be included. Considering a rejection rate of 20 %, about 1879 patients need to be approached.

### Data management

All the data will be entered and stored in a password-protected computer. To ensure high quality of the data, a double data entry method will be used. A data monitoring committee, of which at least two members will be independent of the research team, will monitor the data management process regularly. All the data will be frozen and then locked to prevent further editing after the validation by the data monitoring committee. Only the data monitoring committee, the study research assistant and the principal investigator will have access to the final data set. The protocol and statistical results will be published in a scientific journal.

### Statistical analysis

All analyses will be performed based on the intention-to-treat principle with differences assumed to be significant at a two-sided *P* value < 0.05.

Baseline characteristics and follow-up information will be collected for each group through the questionnaire. Absolute values for each question will be used to calculate the mean value, standard deviation, median value, percentile, number of cases, and percentages per group. Statistical significance will be evaluated by analysis of variance or Kruskal–Wallis rank sum test for continuous variables and chi-squared test for categorical variables, respectively. Kaplan–Meier curves will be used to describe the time-dependent occurrence of events, and the log-rank test will be performed to compare survival distributions for the four groups. To adjust for possible baseline imbalances between groups, a Cox proportional hazards model will be used. A hazard ratio will be calculated. In addition, subgroup analyses will be performed corresponding to the nature of the data. All statistical analyses will be performed using SPSS statistical software (version 17.00, Chicago, IL, USA).

### Quality assurance

#### Steering committee

The intervention of the steering committee includes general practice training and practice visits, to develop and monitor the implementation of the protocol. Telephone support is delivered by the intervention team with assistance from the principle investigators. The quality of the intervention process will be monitored and assured by a steering committee using multiple strategies, including a standardized selection, training and performance assessments of the intervention team, evaluation of general practice training, records of practice visits kept by the intervention team, and ongoing feedback by practice staff on the intervention during the 24-month period. The steering committee will be supported by a statistician, who is responsible for ensuring the timely publication of this study results.

#### 7-day Holter diagnosis committee

This committee will determine the rhythm of the 7-day Holter ECGs without knowing the patients’ conditions and will be responsible for the diagnosis of the recorded 7-day Holter ECGs before the analysis of the primary and secondary outcome measures.

#### Safety monitoring board

The safety monitoring board will be responsible for monitoring patient safety and will recommend premature cessation of the trial should there be an increase in unpredicted adverse events.

## Discussion

Paroxysmal AF often progresses to persistent or permanent AF when the former increases in frequency and duration, and accounts for approximately 5.5 % of all patients with paroxysmal AF per year [17]. One consistent fact that emerges from studies is that conventional anti-arrhythmic agents, such as class I antiarrhythmics, do not improve survival rates [18], and their efficacy in preventing progression of paroxysmal AF to persistent AF is limited because they do not exert any potential benefits on electrical or structural remodeling, which contributes to the pathophysiological basis of AF [19].

Preventing new-onset and recurrent AF with upstream therapies is of great interest, but current data are conflicting. Further studies are needed to optimize rhythm control by anti-arrhythmic drugs and targeted catheter ablation to specific patient populations at an earlier stage. There is little data on valsartan and fluvastatin treatment in patients with hypertension and non-permanent AF.

### Stains: fluvastatin

During recent years, statins have emerged as one of the most effective treatments to reduce the burden of cardiovascular disease worldwide. Owing to their remarkably good safety profile and declining costs, there has been some interest in the potential use of statins as direct anti-arrhythmic or anti-inflammatory drugs [20]. Meta-analysis demonstrated that the use of statins was significantly associated with a decreased risk of AF in patients with sinus rhythm [12, 15, 21, 22]. Statins could be considered for patients with intermediate risk factors [23] as a secondary prevention of AF [22]. These results provided some evidence for the benefit of statins beyond their lipid-lowering activity [20].

The potential mechanisms involved in AF reduction associated with statin therapy are not fully clear. One possible pathway involves inflammation, which has been recognized as an accomplice and a potential trigger for AF [24]. Bellosta *et al.* [25] have suggested that statins have pleiotropic properties, and their anti-inflammatory effects are associated with a reduction in the expression of cytokines, intercellular adhesion molecules, and interleukins. Statins reduced the incidence of paroxysmal AF with a concomitant decrease in C-reactive protein levels [26], which are believed to be a risk factor for AF. Statin treatment reduced inflammatory biomarkers, which might also explain a potentially beneficial effect of statins against AF [27]. More recently, antioxidative actions have been hypothesized to prevent electrical remodeling [28]. The administration of statins significantly decreased generation of reactive oxygen species *in vitro* and *in vivo* [29]. Statins prevented the development of AF by modulating extracellular remodeling. Statins modified extracellular components by regulating the expression of matrix metalloproteinases or their inhibitors [30]. Moreover, statins might downregulate the RAS and modulate autonomic nervous system-induced increases in sympathetic activity, which has been shown to promote atrial remodeling. Other potential mechanisms of action include modification of atherosclerotic plaque [31], improvement of endothelial function [32], and alteration of membrane fluidity and ion channel conductance [21].

Some clinical and experimental studies have suggested the use of statins to protect against AF [33]. However, insufficient data are available to allow the recommendation of statins for the prevention of AF [34]. The STOP AF trial [27] showed that high-dose atorvastatin did not reduce the recurrence of AF after cardioversion. Lee *et al.* [35] have suggested that statin therapy in patients with paroxysmal AF might be limited to the prevention of incident AF, but it does not appear to inhibit the progression of paroxysmal AF to permanent AF.

Although some studies [36] established diverse results, they concur that statins are beneficial in decreasing the frequency of paroxysmal AF. We are, however, unable to conclude whether a benefit exists with the use of statins in inhibiting the progression of paroxysmal to persistent AF, owing in part to the limited data available.

Interestingly, fluvastatin was able to confer benefit, despite having the lowest potency. An advantage of fluvastatin is its long-acting formation, which the DECREASE study investigators stated could serves as a ‘bridge’ during the post-operative period, when patients were not receiving oral medications. For practice to reflect clinical trials, atorvastatin would be selected before percutaneous coronary intervention and coronary artery bypass graft, whereas fluvastatin would be selected before noncardiac surgical procedures [13]. Therefore, we selected fluvastatin as the treatment statin in our study. Because it is not clear whether the effects of statin use on AF depend on the underlying lipid levels, we will enroll patients without severe hyperlipidemia.

### ARBs: valsartan

The VALUE trial [3] found that the incidence of persistent AF was 1.35 % with valsartan and 1.97 % with amlodipine (unadjusted hazard ratio 0.683, 95 % confidence interval: 0.525–0.889, *P* = 0.0046). The VALUE trial results demonstrated that valsartan-based treatment reduced the development of new-onset AF, particularly sustained AF in hypertensive patients, compared with amlodipine-based therapy. These findings suggest that ARBs might result in greater benefits than calcium antagonists in hypertensive patients at risk of AF. This leads to the question of whether or not the RAS is an underlying mechanism that can be targeted to prevent AF. Several mechanisms have been proposed to explain the effects of RAS blockade on prevention of AF, including decreased atrial stretch, lowered end-diastolic left ventricular pressure and left atrial pressure, prevention of atrial fibrosis, modification of sympathetic tone, alteration in potassium currents and atrial refractoriness, and direct anti-arrhythmic effects [37–40]. However, a recent J-RHYTHM II study [11] has found that there were no significant differences between an ARB (candesartan) and a CCB (amlodipine) in the development of persistent AF in the treatment of paroxysmal AF associated with hypertension.

Therefore, large-scale, prospective, randomized clinical trials are urgently needed to establish whether statins or ARB bring a similar benefit and are an appropriate therapeutic option in the hypertensive patients for the management of AF.

### 7-day Holter

The Holter monitor has been recently developed, which can perform ECG continuously without the need for removal during exercise or sleeping. However, the 24-hour Holter cannot record arrhythmia for longer than one day. To monitor AF consistently, we used the 7-day Holter system and patients’ diaries to record AF in a Chinese population.

The VF-HT-AF study is a prospective, randomized, open-labeled, multicenter trial designed to provide novel data necessary to comprehensively assess the efficiency of valsartan and fluvastatin on AF prevention in patients with hypertension and non-permanent AF.

### Expected implications

The VF-HT-AF study will determine the anti-arrhythmic effects of the ARB and statin on the recurrence of non-permanent AF associated with hypertension. It will also examine the mortality, morbidity, and perpetuation of the arrhythmia under valsartan, fluvastatin, dihydropiridine CCBs or combined treatment.

In particular, prevention of persistent and permanent AF would be expected to reduce hospitalization for heart failure, the incidence of stroke, morbidity, and mortality in patients with hypertension. This study will provide data to health care policymakers and the authors of clinical guidelines regarding the appropriate use of upstream therapy in hypertensive patients with AF and provide directions for future research.

Therefore, the results of this study will emphasize the role of upstream therapies in the prevention of AF in a Chinese hypertensive population and assist in the design of an optimal therapy for such patients.

However, one important limitation that might be present in this study is that it is an open-label trial, because we do not have enough funds to make the drugs in blinded versions. The drugs that the patients will use in our study are all provided in hospitals or pharmacies. The patients and doctors will know the drugs used. Another limitation is that the control group is not blank, so we do not test the fluvastatin effect alone. Because the patients in our study are hypertensive patients, they must, for ethical reasons, be treated with antihypertensive therapy. We chose the group receiving dihydropyridine CCBs as the control group, comparing them with the dihydropyridine CCBs plus fluvastatin group. If the recurrence of AF in the dihydropyridine CCBs plus fluvastatin group is less than the dihydropyridine CCBs group, it will be proved that fluvastatin can reduce the probability of non-permanent AF in patients with hypertension. There may be some combined effects of dihydropyridine CCBs plus fluvastatin, but we have to use antihypertensive drugs.

## Trial status

The trial is currently in the recruitment phase.

## Additional files

Additional file 1:
**Ethical bodies that approved the study.**
Additional file 2:
**SPIRIT-Checklist 2013.**
Additional file 3:
**Informed consent statement.**

## Abbreviations

ACEI: angiotensin-converting enzyme inhibitor
AF: atrial fibrillation
ARB: angiotensin II receptor blocker
CCB: calcium channel blocker
CONSORT: Consolidated Standards of Reporting Trials
ECG: Electrocardiography
NYHA: New York Heart Association
RAS: renin-angiotensin system
SPIRIT: Standard Protocol Items: Recommendations for Interventional Trials
