# Supplementary material for: Upstream therapeutic strategies of Valsartan and Fluvastatin on Hypertensive patients with non-permanent Atrial Fibrillation (VF-HT-AF): study protocol for a randomized controlled trial
Source: Trials. 2015 Aug 7;16:336. doi: 10.1186/s13063-015-0836-5 (PMC4528391; doi:10.1186/s13063-015-0836-5)
Supplement: Additional file 1: — Ethical bodies that approved the study. [file 13063_2015_836_MOESM1_ESM.doc]

**Additional file1:**

**The list of the names of all ethical bodies that approved our study in the various centres involved**

Second Hospital of Tianjin Medical University

First Hospital of Tianjin University of Traditional Chinese Medicine

Second Hospital of Tianjin University of Traditional Chinese Medicine

Tianjin Academy of Traditional Chinese Medicine Affiliated Hospital

Tianjin 4^th^ Centre Hospital

Tianjin Chest Hospital

Tianjin Port Hospital

Jinghai District Hospital in Tianjin

Dongli District Hospital in Tianjin

Tianjin Beichen District Chinese Medicine Hospital

The First Department of Cardiology in People's Hospital of Tianjin

The Third Department of Cardiology in People's Hospital of Tianjin

First Hospital of Qinhuangdao

The First Department of Cardiology in Tangshan Gongren Hospital

The Third Department of Cardiology in Tangshan Gongren Hospital
