# Supplementary material for: Upstream therapeutic strategies of Valsartan and Fluvastatin on Hypertensive patients with non-permanent Atrial Fibrillation (VF-HT-AF): study protocol for a randomized controlled trial
Source: Trials. 2015 Aug 7;16:336. doi: 10.1186/s13063-015-0836-5 (PMC4528391; doi:10.1186/s13063-015-0836-5)
Supplement: Additional file 3: — Informed consent statement. [file 13063_2015_836_MOESM3_ESM.doc]

**Additional file 3. Informed consent statement**

**INFORMED CONSENT STATEMENT**

**Upstream therapeutic strategies of** **Valsartan and Fluvastatin on** **Hypertensive patients with** **non-permanent Atrial Fibrillation (****VF-HT-AF): a randomized controlled trial**

You are invited to participate in a research study. Before you decide to take part in this study, please make sure you have fully understood the purpose as well as important related things about the research. Please think it deeply before your decision.

**INFORMATION**

Atrial fibrillation (AF) is associated with increased mortality, especially in hypertensive patients. In general, hypertension is the most important risk factor for AF. In the last several years, a number of trials investigating upstream therapy for prevention of AF have been reported, such as valsartan and fluvastatin. However, it still requires rigorous scientific evidence from clinical trials that show direct correlation. Hence, the purpose of this study is to evaluate the efficacy of the upstream therapeutic strategies of valsartan and/or fluvastatin on hypertensive patients with non-permanent AF by a randomized controlled clinical trial.

**WHO IS SUITABLE?**

You need to meet the inclusive recruited criteria: (1) hypertension, defined as an average systolic blood pressure (BP) ≥ 140 mmHg and/or a diastolic BP ≥90 mmHg (but a systolic BP < 180 mmHg and a diastolic BP < 110 mmHg) of the first visit, or requiring any anti-hypertension treatment at enrollment; (2) a history of non-permanent AF within 1 year prior to the enrollment, which has been confirmed by electrocardiograms (ECG) , and converted to sinus rhythm; (3) those who haven’t taken ARBs and/or angiotensin-converting enzyme inhibitors (ACEIs) as well as statins in the past 2 weeks, or those who are having ARBs, ACEIs and statins, can accept 2 weeks washout period ; (4) ages ranged from 25 to 79 years; and (5) signed the informed consent.

You are not suitable if you have the following condition: (1) persistent AF with a duration ≥1 year, and/or permanent AF; (2) serious left main coronary artery disease identified by coronary angiography; (3) heart failure [New York Heart Association (NYHA) III or IV]; (4) acute myocardial infarction in 3 months; (5) with surgical/interventional indications of valvular heart disease; (6) uncontrolled [thyroid](app:ds:thyroid) [disease](app:ds:disease) (abnormal free T3, free T4, or thyroid stimulating hormone, or requiring any anti-thyroid treatment at enrollment); (7) serious liver/renal dysfunction (ALT > 80 u/L and/or AST > 80 u/L, and/or creatinine > 132 umol/L); (8) history of unstable angina pectoris; (9) stroke or transient ischemic attack (TIA) history within the past 3 months; (10) poor treatment compliance, such as the central nervous system/mental illness, or may not be cooperative in the follow-up period; (11) patients have had ARBs and/or ACEIs and/or statins but can’t accept the washout period for 2 weeks; (12) patients with obvious hyperlipidemia must be treated by statins and/or fibrates; (13) contraindication of statins and/or ARBs; and (14) pregnancy or the possibility of pregnancy, or breast feeding.

**HOW TO DO?**

If you are suitable and did not take ARBs, ACEIs and/or statins in the past at least 14 days, you will be directly enrolled and randomized to one of four groups（the valsartan group, the fluvastatin plus dihydropyridine CCBs group, the valsartan plus fluvastatin group, and the dihydropyridine CCBs group）. If you have taken ARBs, ACEIs and/or statins, you will be randomized after 14 days washout period.

The clinical follow-up will be performed every 3 months during the 2 years follow-up period. At the baseline, 3^rd^ month and the end of the follow-up period, participants may have blood examinations to ensure normal function as well as the safety of the research. Ultrasound echocardiography will be performed before the patients enter the study and at the end of the follow-up period. A 7-day Holter monitoring will be performed at the baseline, 6^th^ month, 12^th^ month and at the end of the follow-up period. Patients’ diaries, cardiac function of NYHA classification, adverse events during the follow-up will be collected every three months.

Throughout the study, you should record diaries when you feel discomfort. The attending physicians will also record BP and ECG during each follow-up. Unless it is necessary, antiarrhythmic drug therapy will be discontinued during the study according to the attending physicians’ advice.

**RISK**

There are commercial medicines in this study. However, there may be some side-effect. A small number of people may experience adverse events such as edema, constipation, headache, gastrointestinal symptom, hepatitis, myopathy and the like. In case it happens, the clinical trial will be stop immediately and further medical treatment or referral will be given if necessary.

**BENEFITS**

There will be experience physicians who are responsible for you during the 2 years follow-up. You can enjoy the convenience of registration and consult the expert for free. Besides, there will be 4 times free 7-days Holter examination. You will be taught about how to measure blood pressure and electrocardiogram at home. Though taking part in this research may or may not be beneficial the individual, but it will be helpful to find a treatment for hypertensive patients with non-permanent Atrial Fibrillation.

**CONFIDENTIALITY**

Every participant will be assigned a code which can be traced back to the group allocation and the name can not be found on the page of case report form (CRF). CRF, patient diaries and related document will be kept entirely confidential. Only the research team and the ethics committee member have rights to read them. The reports will be written mainly in aggregate term, but individual responses to drug may also be described and no personal data will be disclosed in any report. All the information provided by the participant will be kept confidential by the research team.

**COMPENSATION**

If you get any other disease during the research period, no compensation will be provided for it. If you have direct injury due to the research, you can apply for compensation with enough and strong evidence.

**PARTICIPATION**

You are free to withdraw from this study at any time. Your decision to withdraw will have no effect on any other service or program provided to you. If you decide to withdraw from the study, you may be asked some information about your condition.

**RESEARCH UNIT** ___________________

**CONSENT**

I have read and understand the above information and given the chance if I question. I agree to participate in this study and would be given a copy of this form.

＿＿＿＿＿＿＿＿＿＿＿＿＿ ＿＿＿＿＿＿＿＿＿＿＿＿＿ ＿＿＿＿＿＿＿＿＿＿＿

Signature of participant/proxy Date Telephone

＿＿＿＿＿＿＿＿＿＿＿＿＿ ＿＿＿＿＿＿＿＿＿＿＿＿＿ ＿＿＿＿＿＿＿＿＿＿＿

Signature of investigator Date Telephone

－End of Informed Consent Statement－
